# Development of Phage Cocktails to Treat *E. coli* Catheter-Associated Urinary Tract Infection and Associated Biofilms

**DOI:** 10.3389/fmicb.2022.796132

**Published:** 2022-05-10

**Authors:** Belkys C. Sanchez, Emmaline R. Heckmann, Sabrina I. Green, Justin R. Clark, Heidi B. Kaplan, Robert F. Ramig, Kenneth L. Muldrew, Casey Hines-Munson, Felicia Skelton, Barbara W. Trautner, Anthony W. Maresso

**Affiliations:** ^1^Tailored Antibacterials and Innovative Laboratories for Phage (Φ) Research, Department of Molecular Virology and Microbiology, Baylor College of Medicine, Houston, TX, United States; ^2^Department of Microbiology and Molecular Genetics, McGovern Medical School, UTHealth Houston, Houston, TX, United States; ^3^Department of Pathology and Immunology, Baylor College of Medicine, Houston, TX, United States; ^4^Pathology and Laboratory Medicine, Michael E. DeBakey VA Medical Center, Houston, TX, United States; ^5^Section of Infectious Diseases, Department of Medicine, Baylor College of Medicine, Houston, TX, United States; ^6^Center for Innovations in Quality, Effectiveness and Safety, Michael E. DeBakey VA Medical Center, Houston, TX, United States; ^7^H. Ben Taub Department of Physical Medicine and Rehabilitation, Baylor College of Medicine, Houston, TX, United States; ^8^Department of Medicine and Surgery, Baylor College of Medicine, Houston, TX, United States; ^9^Center for Translational Research on Inflammatory Diseases, Michael E. DeBakey VA Medical Center, Houston, TX, United States

**Keywords:** phage therapy, uropathogenic *E. coli*, multidrug-resistance, CAUTI, biofilms

## Abstract

High rates of antimicrobial resistance and formation of biofilms makes treatment of *Escherichia coli* catheter-associated urinary tract infections (CAUTI) particularly challenging. CAUTI affect 1 million patients per year in the United States and are associated with morbidity and mortality, particularly as an etiology for sepsis. Phage have been proposed as a potential therapeutic option. Here, we report the development of phage cocktails that lyse contemporary *E. coli* strains isolated from the urine of patients with spinal cord injury (SCI) and display strong biofilm-forming properties. We characterized *E. coli* phage against biofilms in two *in vitro* CAUTI models. Biofilm viability was measured by an MTT assay that determines cell metabolic activity and by quantification of colony forming units. Nine phage decreased cell viability by >80% when added individually to biofilms of two *E. coli* strains in human urine. A phage cocktail comprising six phage lyses 82% of the strains in our *E. coli* library and is highly effective against young and old biofilms and against biofilms on silicon catheter materials. Using antibiotics together with our phage cocktail prevented or decreased emergence of *E. coli* resistant to phage in human urine. We created an anti-biofilm phage cocktail with broad host range against *E. coli* strains isolated from urine. These phage cocktails may have therapeutic potential against CAUTI.

## Introduction

Urinary tract infections (UTI) are among the most common community and nosocomial bacterial infections (Flores-Mireles et al., 2019; Medina and Castillo-Pino, 2019) affecting 150 million people worldwide each year (Stamm, 2002), and resulting in a high economic burden on society (Lo et al., 2014; Skelton et al., 2019). UTI cause serious complications, including pyelonephritis, sepsis and frequent recurrences, resulting in repetitive antimicrobial administration and selection of multidrug-resistant uropathogens (Flores-Mireles et al., 2019). The presence of a urinary catheter facilitates entrance and colonization of pathogens to the urinary tract, increasing the risk of infection (Jacobsen et al., 2008). Although efforts have been made to improve prevention and management of catheter-associated urinary tract infections (CAUTI), almost all long-term catheterized patients develop bacteriuria, and 10–30% of patients with catheter-associated bacteriuria develop UTI-related symptoms (Warren, 1991; Trautner and Darouiche, 2004). CAUTI is one of the most common infections acquired in hospitals, accounting for 40% of all nosocomial infections and 1 million cases in the United States each year (Tambyah and Maki, 2000; Stamm and Norrby, 2001; Foxman, 2010). Persons with spinal cord injury (SCI) suffer disproportionately from CAUTI, given the secondary complication of neurogenic bladder and the need for chronic urinary catheterization (Manack et al., 2011; Skelton et al., 2015; Skelton-Dudley et al., 2019). Persons with SCI also experience a higher proportion of multidrug-resistant infections due to frequent healthcare exposure and courses of antibiotics over their lifetime (Kang et al., 2015; Suda et al., 2016; Evans et al., 2017). CAUTI is associated with increased morbidity and mortality in persons with SCI and the management of this condition presents unique challenges in this patient population (Skelton-Dudley et al., 2019).

*Escherichia coli* is the main causative agent of UTI, causing 80% of acute UTI and 33% of CAUTI (Stamm, 2002; Stickler, 2008; Foxman, 2010). Similarly, *E. coli* is one of the most commonly isolated pathogens from the urine of persons with SCI (Kang et al., 2015). *E. coli* can form biofilms on urinary catheters which complicates management of CAUTI (Stickler, 2008; Niveditha et al., 2012). Biofilms are surface-associated multicellular bacterial communities that protect individual cells from host defenses and environmental stresses, and mediate bacterial persistence and recurrent infections in the urinary tract (Trautner and Darouiche, 2004; Soto et al., 2006). Due to their structural and metabolic properties, biofilms are recalcitrant to antimicrobial therapy (Hall and Mah, 2017). Many antibiotics do not easily kill cells within biofilms (Singh et al., 2016; Ciofu et al., 2022), and *E. coli* biofilms have been observed on urinary catheters recovered from patients that received antibiotic therapy (Walker et al., 2020). Furthermore, persister cells within the biofilm can reemerge once antibiotic therapy is discontinued (Gollan et al., 2019). Thus, not only do antibiotics often fail to eradicate biofilms, but repetitive therapy required to treat recurrent UTI can select for resistant microorganisms.

The current approach for treatment of CAUTI includes targeted antibiotic therapy and replacement of the indwelling catheter (Hooton et al., 2010; Fekete, 2021), which may contain biofilms of the infecting organism (Trautner and Darouiche, 2004). Because multidrug-resistant UTI represent a threat to the health and quality of life of persons with SCI, a new management approach is needed. To address this unmet need, here we have characterized bacteriophage (phage or Φ) with specificity toward contemporary *E. coli* strains isolated from the urine of patients with SCI. Phage are ubiquitous viruses that infect and kill bacteria irrespective of their antibiotic sensitivity (Chan et al., 2013). Phage have been successfully used to treat biofilm-associated infections recalcitrant to antibiotics (Wright et al., 2009; Chan et al., 2018; Aslam et al., 2019, 2020; Cano et al., 2020). Additionally, phage may self-dose (Terwilliger et al., 2020), be evolved to re-target phage-resistant strains (Salazar et al., 2021) and have features that enhance their activity in the complex microenvironments of the mammalian host, especially at mucosal surfaces (Green et al., 2021). Our group previously characterized a library of phage that lyse multidrug-resistant extra-intestinal pathogenic *E. coli* strains (Gibson et al., 2019). Some of these phage have been shown efficacious in several murine models of infection and in a case of compassionate use of phage to treat a recurrent UTI (Green et al., 2017; Terwilliger et al., 2021). Here, we screened and characterized this phage library and additional novel phage for their ability to reduce the viability of bacterial cells in biofilms of *E. coli* clinical strains. Our data reveals that it is possible to generate highly lytic cocktails with anti-biofilm activity against most *E. coli* isolates from our population of patients with SCI, and that these cocktails are active against biofilms grown in human urine and on silicone catheter materials.

## Materials and Methods

### Collection and Storage of *E. coli* Clinical Isolates

De-identified *E. coli* strains isolated from urine specimens of patients with SCI and their antibiotic susceptibility data were obtained from the Clinical Microbiology Laboratory at the Houston Veterans Administration, with approval of the Baylor College of Medicine Institutional Review Board (Protocol H-29737). One isolated colony of each strain was grown overnight in LB medium, diluted 1:10 into LB medium containing 15% glycerol, and frozen at −80°C, until later use.

### Bacteriophage

The phage used here were either previously characterized (Gibson et al., 2019) or newly isolated from wastewater by plaque assay. Plate stocks of single phage were used for the anti-biofilm phage screens. Phage were purified by cesium chloride gradient centrifugation (Green et al., 2017) for further characterization and preparation of phage cocktails.

### Human Urine Collection and Processing

Eight healthy donors (four females and four males) collected urine over 24–48 h. All urine samples were combined in equivalent volumes, filtered (0.22 μm pore size) and aliquots stored at 4°C. Use of human urine was approved by the Baylor College of Medicine Institutional Review Board (Protocol H-29737).

### Biofilm Viability Assay

Viability of cells within biofilms was determined by an MTT assay (Mapes et al., 2016; Grela et al., 2018) with modifications. Overnight cultures of *E. coli* were diluted (1:100) in tryptic soy broth (TSB) and seeded in 96-well tissue culture treated plates (Corning Inc., Corning, NY, United States). Plates were incubated at 37°C for 24 h, washed with phosphate buffered saline (PBS), and incubated with 3-(4,5-dimethylthiazol-2-yl)-2,5-diphenyltetrazolium bromide (MTT) at a concentration of 0.45 mg/mL for 3 h in the dark. Then, the MTT was removed, and the resulting formazan crystals were dissolved with dimethyl sulfoxide (DMSO) for 10 min. The metabolic output of cells within the biofilm was determined by measuring absorbance at 540 nm in a Biotek Synergy HT (BioTek, Winooski, VT, United States). To grow biofilms in urine, overnight cultures of *E. coli* were diluted (1:100) in LB medium and incubated for 2 h. Cells were harvested, washed, and normalized to OD_600_ = 0.03 in urine supplemented with 20 mg/mL of bovine serum albumin (BSA) (Colomer-Winter et al., 2019), seeded in 96-well plates and incubated for 24–48 h or 7 days at 37°C. Fresh BSA-supplemented urine was replenished every 24 h. Biofilm viability was determined as above.

### Phage Treatment of Biofilms on 96-Well Plates

*Escherichia coli* biofilms grown in TSB or human urine supplemented with 20 mg/mL of BSA were washed once with PBS and treated by the addition of phage diluted in the appropriate medium. The plates were incubated for 24 h at 37°C and biofilm viability was determined as described above.

### Phage-Mediated Killing of Planktonic Cells

Overnight cultures were diluted 1:100 in urine and inoculated into untreated 96-well plates (Fisher Bioreagents, Ottawa, ON, Canada) containing phage (final titer of 10^7^ PFU/mL). The OD_600_ was measured every 15 min at 37°C for a total of 24 h with continuous shaking in a BioTek Synergy HT (BioTek, Winooski, VT, United States) plate reader. The results are shown as bacterial cell density and percent of untreated control cell density [% untreated control = (OD_600_ treated × 100)/OD_600_ untreated control] at *t* = 24 h.

### Determination of Host Range and Virulence of Phage

The spot titration protocol described by Gibson et al. (2019) was used to determine phage host range and phage virulence by efficiency of plating (EOP). Phage titers were determined by counting individual plaques at the terminal dilution, and EOP was calculated by dividing the titer of the phage on the test strain by the titer of the same phage on its isolation strain.

### Identification and Comparison of Putative Phage Depolymerase Enzymes

To identify potential depolymerase enzymes, we parsed the RASTtk annotated genomes of phage for keywords related to depolymerases: “lysozyme,” “lysis,” “muramidase,” “hydrolase,” “sialidase,” “levanase,” “xylanase,” “dextranase,” “rhamnosidase,” “lyase,” “hyaluronidase,” “pectin,” “pectate,” and “lipase” (Aziz et al., 2008; Overbeek et al., 2014; Brettin et al., 2015; Pires et al., 2016; Latka et al., 2017; Knecht et al., 2019). We also annotated the domains of each genome using the United States Department of Energy Systems Biology Knowledgebase (KBase) “Annotate Domains in a Genome” app (v1.0.7), which annotates domains using RPS-BLAST (v2.2.31) with COGs (v3.16), CDD (v3.16), SMART (v6.0), and PRK (v6.0), and HMMER (v3.1b2) with Pfam (v31.0), TIGRFAMs (v15.0), and NCBIfam (v1.1) (Arkin et al., 2018). These results were parsed using the same keywords described above.

Alignments were performed using Geneious Alignment in Geneious Prime 2022.0.2^1^ or MAFFT (v7.450) (Katoh et al., 2002; Katoh and Standley, 2013). Literature searches were accomplished using keywords and using translations of putative depolymerase enzymes in PaperBLAST (Price and Arkin, 2017).

### Evaluation of Phage-Antibiotic Interactions

Synography, a method to measure phage and antibiotic synergy using an optically based microtiter plate readout was performed as previously described (Gu Liu et al., 2020). A normalized bacterial suspension (OD_600_ = 1) in urine was seeded into untreated microtiter plates (Fisher Bioreagents, ON, Canada) containing a checkerboard of phage and antibiotic concentrations. The OD_600_ was measured every 15 min at 37°C for a total of 24 h with continuous shaking. Antibiotic stocks were prepared as follows: ciprofloxacin hydrochloride (Corning Inc., Christiansburg, VA, United States) was dissolved in ddH_2_O, and trimethoprim and sulfamethoxazole (Sigma-Aldrich, St. Louis, MO, United States) was dissolved in DMSO (final <1%).

### *Escherichia coli* Biofilm Growth on Silicon Catheter Materials and Phage Treatment

Sterile pieces of RenaSil (0.012 inches inner diameter, 0.025 inches outer diameter, 6 mm length) silicone tubing were suspended in 3 mL of normalized bacterial suspensions (OD_600_ = 0.03) in urine supplemented with 20 mg/mL of BSA and incubated for 48 h at 37°C with shaking. Then the silicone tubing pieces were washed, transferred to 1.5 mL microfuge tubes containing phage cocktails in urine, and incubated for 4–6 h at 37°C with shaking. Subsequently, the silicone tubing pieces were washed, transferred to a tube with 1 mL of PBS, vortexed, sonicated for 5 min in a water bath and vortexed again. The supernatant was serially diluted and plated to quantify the adherent cells.

### Statistical Analysis

Statistical analysis was performed with GraphPad PRISM 8 software using one-way ANOVA or two-way ANOVA, unless stated otherwise. Dunnett’s test was performed for multiple comparisons. All graphs show the mean and standard deviations of at least three biological replicates from at least two independent experiments.

## Results

### Assessment of Biofilms From *E. coli* Clinical Isolates

**FIGURE 1:**
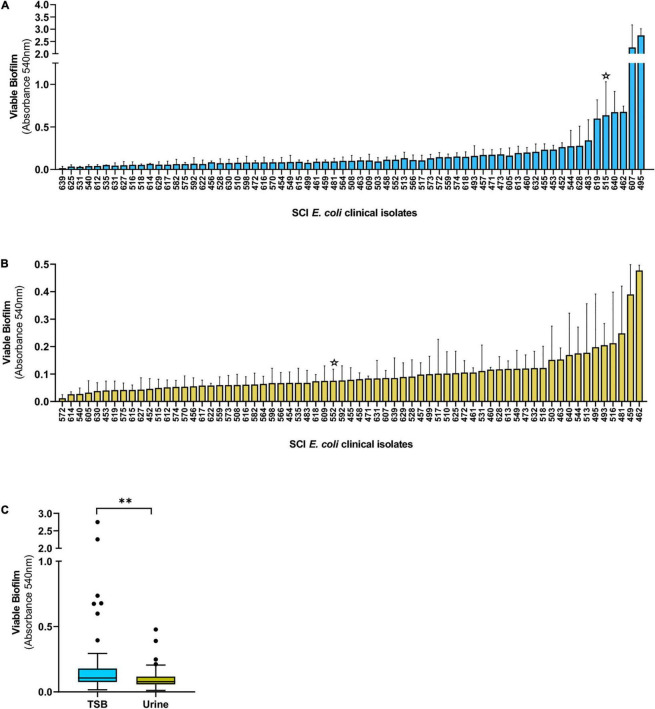
Biofilm formation by *E. coli* isolated from the urine of patients with SCI. Biofilms were grown in TSB **(A)** or human urine **(B)** for 24 h and the levels of viable biofilm determined with an MTT assay. **(C)** Tukey’s style box and whiskers plot summarizing viable biofilm levels of all *E. coli* strains depicted in **(A,B)**. The stars indicate isolates DS515 and DS552 used for phage screenings in Figure 2. ^**^p < 0.01 determined by Mann–Whitney test.

We assessed biofilm formation by *E. coli* strains isolated from the urine of patients with SCI at the Houston Veteran Affairs Hospital from October 2018 to December 2019. Biofilms established in TSB or urine were washed and treated with MTT to determine their metabolic output. We found a high prevalence of biofilm formation by SCI *E. coli*, with 98% (66/67) and 100% of isolates forming biofilms in TSB and urine, respectively (Figures 1A,B). Median biofilm levels in TSB were higher compared to urine (0.1065 versus 0.0789, Mann–Whitney test, *p* = 0.002) (Figure 1C). The spread of the values was more uniform for biofilm formation in urine, while outliers were observed over a larger range of absorbances in TSB. Biofilm formation among individual strains varied in both media with only 4 of the top 10 biofilm formers in TSB (495, 462, 640, and 544) also forming high levels of biofilms in urine.

### Identification of Phage With Lytic Activity Against *E. coli* Biofilms

**FIGURE 2:**
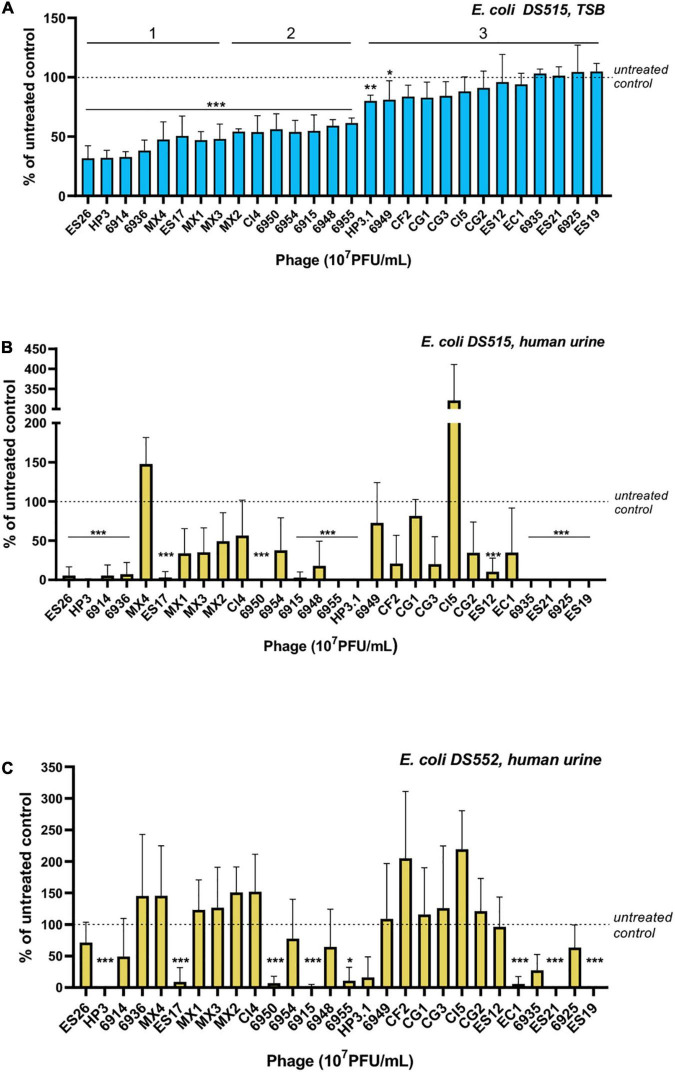
Screens of *E. coli* phage for anti-biofilm activity. Viability of cells in the biofilm under each condition after 24-h treatment with 10^7^ PFU/mL of phage is represented as a percentage of the untreated control. *E. coli* DS515 biofilms in TSB **(A)** and human urine **(B)**, and *E. coli* DS552 biofilms in human urine **(C)** are shown. Phage were classified into groups 1, 2, and 3, based on their high, intermediate, or low/no anti-biofilm activity. ***p < 0.001, **p < 0.01, *p < 0.05.

We hypothesize that phage that reduce *in vitro* biofilms will have therapeutic potential to treat CAUTI and its associated biofilms. Thus, we screened phage (Supplementary Table 1) for their ability to reduce *E. coli* grown in biofilms in TSB and urine. *E. coli* DS515 was selected for this screen due to its ability to form robust biofilms (star, Figure 1A). Of the 28 phage tested against DS515 biofilms grown in TSB, 8 (28.6%, group 1) decreased biofilm viability by >50%, 7 (25%, group 2) reduced biofilm viability by 25–50%, and 13 phage (46.4%, group 3) did not cause a significant reduction in biofilm viability compared to the untreated control (Figure 2A). Interestingly, 53% (8/15) of the phage classified in groups 1 and 2 produced plaques with halos on DS515 lawns (Supplementary Table 1).

Since we observed differences in biofilm formation by *E. coli* in TSB and urine, we tested phage against biofilms formed in urine. To identify phage with broad anti-biofilm activity in urine, phage were tested against two *E. coli* strains, DS515 and DS552. *E. coli* DS552 was an average biofilm former in urine (star, Figure 1B). The phage demonstrated diverse levels of activity against biofilms formed in urine (Figures 2B,C). Treatment with nine phage (HP3, ES17, 6950, 6915, 6955, HP3.1, 6935, ES21, and ES19) resulted in >80% reduction in biofilm viability on both strains. Treatment with some phage (for example CI5, Figure 2B) caused increased biofilm formation compared to the untreated control, particularly in strain DS552 (11/28 phage treatments), suggesting that characteristics inherent to this strain favor biofilm formation when exposed to some phage as it has been discussed before (Hansen et al., 2019).

### Anti-biofilm Phage Kill Planktonic Cells in Human Urine

**FIGURE 3:**
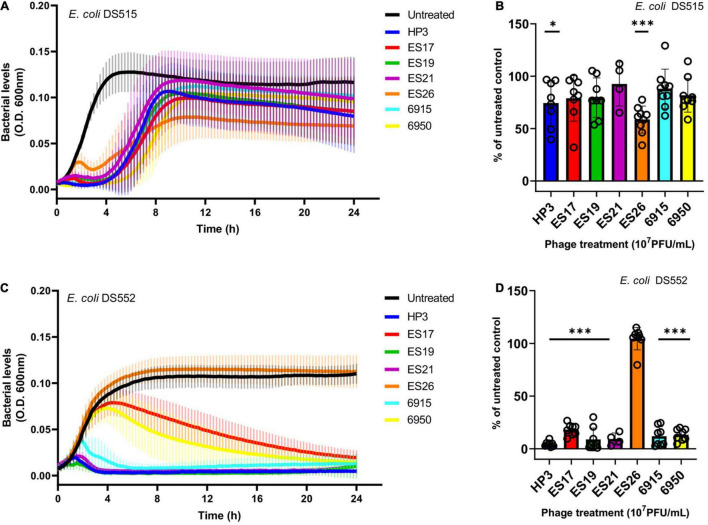
The activity of anti-biofilm phage against *E. coli* planktonic cells in human urine. Bacterial growth was assessed for 24 h in the presence or absence of phage (10^7^ PFU/mL). Bacterial growth was determined by OD_600_ over time and as a percentage of the untreated control at 24 h, respectively, for *E. coli* DS515 **(A,B)** and *E. coli* DS552 **(C,D)**. ***p < 0.001, *p < 0.05.

The biofilm life cycle is dynamic and includes adherent and planktonic stages. Thus, a subset of phage with high biofilm killing activity (HP3, ES17, ES19, ES21, ES26, 6915, and 6950) was evaluated against planktonic cultures in urine. Phage were added to bacterial suspensions in urine and growth was followed over 24 h (Figures 3A,C). Although most phage tested caused a strong initial suppression of growth and prevented full recovery of the bacterial population, the sensitivity of planktonic cells to phage was strain dependent. All phage except ES26 significantly decreased DS552 levels (Figures 3C,D). In contrast, DS515 showed substantial growth recovery against each phage (Figure 3A). Only treatment with phage HP3 and ES26 caused a significant decrease in DS515 levels at 24 h (Figure 3B). Importantly, phage HP3 killed planktonic cells of both strains. Overall, these results demonstrate that phage HP3, ES17, ES19, ES21, ES26, 6915, and 6950 effectively reduce biofilm viability and are virulent against planktonic *E. coli* in urine, though their activity is strain dependent in the latter.

### Phage and Antibiotic Susceptibility of *E. coli* Clinical Isolates

**FIGURE 4:**
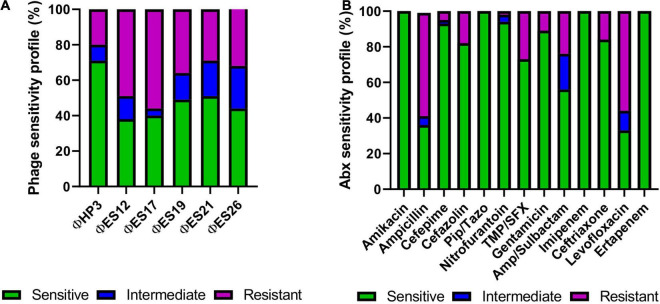
Phage lysis phenotypes and antibiotic sensitivities of 54 strains of *E. coli* isolated from the urine of patients with SCI. **(A)** Susceptibility of SCI *E. coli* strains (*N* = 54) based on phage virulence (EOP) against each strain. **(B)** Antibiotic susceptibilities of SCI *E. coli* strains (*N* = 54, except for Pip/Tazo *N* = 46).

We next examined the host range of promising phage against SCI *E. coli* by using efficiency of plating as a quantitative readout of lytic activity (Gibson et al., 2019; Supplementary Table 2). Figure 4 demonstrates a summary of these results for 54 SCI *E. coli* isolates, including antibiotic susceptibilities for comparison. The analysis of susceptibility to individual phage showed that 38–71% of SCI *E. coli* strains were killed at EOP > 0.001 and were classified as sensitive, whereas 4–24% of strains were killed at 0 < EOP < 0.001 and were classified as having intermediate susceptibility to phage. Ten bacterial strains (10/54) were resistant to all phage (Supplementary Table 2). We observed that 20% of phage-resistant strains were multidrug-resistant (Magiorakos et al., 2012), compared to 55.6% of phage-sensitive strains (Supplementary Table 2). However, no statistically significant association was found between resistance to phage and resistance to multiple antibiotics (Fischer’s Exact test, *p* = 0.0078).

### Design of Anti-biofilm Phage Cocktails to Treat Catheter-Associated Urinary Tract Infections

**FIGURE 5:**
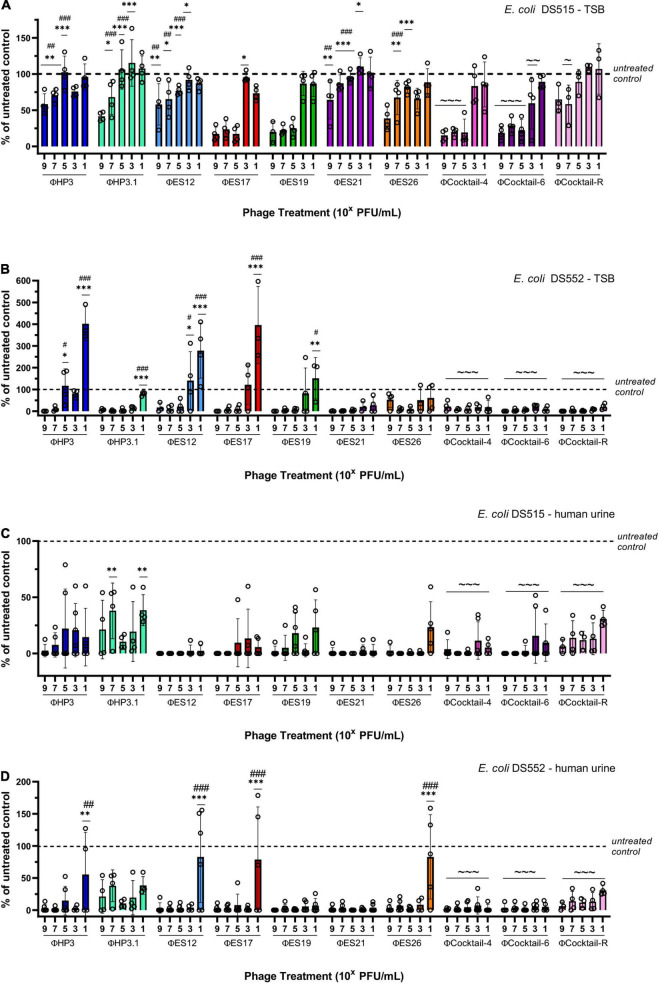
Design of anti-biofilm phage cocktails. Results depict the percentage of viable biofilms after 24-h treatment with phage, using the untreated control biofilm as the denominator. Biofilms of two strains of *E. coli* (DS515 and DS552) were treated with increasing doses of individual phage and three phage cocktails (ΦCocktail-4: HP3, ES17, ES21, and ES26; ΦCocktail-6: HP3, ES12, ES17, ES19, ES21, and ES26; ΦCocktail-R: HP3, ES17, ES19, and HP3.1). Biofilms were grown in TSB **(A,B)** and human urine **(C,D)**. Symbols denote significant difference; * compared to corresponding treatment with ΦCocktail-6, # compared to corresponding treatment with ΦCocktail-4, and ∼ compared to the untreated control. */#/∼*p* < 0.05, ^**^/##/∼∼*p* < 0.01, ^***^/###/∼∼∼*p* < 0.001.

We aimed to design phage cocktails to be highly effective at reducing biofilms and to have broad host range against our library of SCI *E. coli*. The phage used in the cocktails were prioritized based on their ability to reduce biofilms and lyse planktonic cells of *E. coli*. A cocktail composed of four phage (ΦCocktail-4: HP3, ES17, ES21, and ES26) was created, which was capable of lysing 71% of the isolates in our *E. coli* library based on efficiency of plating for each individual phage. To increase the host range of the anti-biofilm phage cocktail, two additional phage were added (ES12 and ES19) to create ΦCocktail-6, which targets 82% of all strains in our *E. coli* library. Biofilms of DS515 and DS552 were treated with increasing concentrations of individual phage or phage cocktails made with equal amounts of each phage. Both phage cocktails reduced viability of biofilms formed in TSB by 81–99.5% (Figures 5A,B), whereas viability of biofilms in urine was reduced by more than 94% compared to the untreated controls (Figures 5C,D). Phage ES17 and ES19 seemed to drive most of the anti-biofilm activity of the cocktails at high concentrations (10^7^–10^9^ PFU/mL) when tested in DS515 biofilms (Figure 5A). In contrast, most individual phage were effective at reducing viability of DS552 biofilms formed in TSB (Figure 5B) at a wider concentration range. Phage activity was more pronounced in urine, where most phage were effective at reducing viability of both strains across all concentration ranges. Both cocktails maintained anti-biofilm activity at lower concentrations compared to individual phage, suggesting there is a synergistic effect of phage within the cocktails. Furthermore, the anti-biofilm activity of ΦCocktail-4 and ΦCocktail-6 was demonstrated against three additional *E. coli* strains (DS457, DS517, and DS549) isolated from the urine of patients with SCI (Supplementary Figure 1). A significant reduction in the viability of biofilms of these strains grown in TSB and urine was observed after 24-h treatment with 10^7^ PFU/mL of phage cocktails.

We observed that higher levels of biofilms of *E. coli* DS515 grown in TSB remained after phage treatment compared to the other conditions tested (Figure 5A). We hypothesized that selection of DS515 phage-resistant cells may be responsible for this phenotype. Thus, we created ΦCocktail-R (HP3, ES17, ES19, and HP3.1) containing a novel phage, HP3.1, which was evolved from phage HP3 and is capable of lysing phage-resistant cells (Salazar et al., 2021). Even though ΦCocktail-R contained phages ES17 and ES19, which independently showed the highest anti-biofilm activity, treatment with ΦCocktail-R was inferior to treatment with the other phage cocktails and individual treatment with ES17 and ES19 (Figure 5A). Only treatment with 10^7^ PFU/mL of ΦCocktail-R showed significant difference compared to the untreated control in biofilms of DS515 grown in TSB. This suggested antagonistic interactions of phage HP3.1 with the other phage in the cocktail under these conditions. In contrast, the activity of ΦCocktail-R against biofilms of DS515 in urine and biofilms of DS457, DS517, DS459, and DS552 (Figures 5B–D and Supplementary Figure 1) in TSB and urine was comparable to the activity of ΦCocktail-4 and ΦCocktail-6 (Figures 5B–D and Supplementary Figure 1). Overall, these data suggest that specific characteristics of biofilms of *E. coli* DS515 in TSB may render it tolerant to phage treatment and this will be further explored in future studies.

### Identification of Putative Depolymerase Enzymes in Anti-biofilm Phage

Most of the phage included in the cocktails produced plaques with halos (Supplementary Table 1; Gibson et al., 2019). This phenotype is associated with degradation of bacterial extra-polymeric substance by phage depolymerase enzymes (Pires et al., 2016). Parsing gene annotations for keywords related to depolymerases uncovered two genes in the T4-like phage HP3, HP3.1, ES12, ES19, ES21, and ES26 (Supplementary Table 3). The first gene encodes the baseplate hub central spike (*gp5* gene in phage T4) that hydrolyzes the bacterial cell wall locally to allow the tail tube to inject the phage DNA (Arisaka et al., 2003). The second gene encodes the endolysin (*e* gene in phage T4), which participates in peptidoglycan degradation from within resulting in bacterial cell lysis and release of phage progeny (Schmelcher et al., 2012). This method also uncovered a putative endolysin in phage ES17.

Since gene annotations can easily miss a potential depolymerase, we also annotated the domains of the genomes and parsed the results for the depolymerase keywords (Supplementary File 1). This method uncovered a putative distal long-tail fiber protein (Supplementary Table 3) in phage HP3, HP3.1, ES12, ES19, ES21, and ES26. This putative tail fiber protein contains a “chaperone of endosialidase” and three pyocin knob domains, two domains found in the endosialidase enzyme of phage K1F which is involved in degradation of the *E. coli* K1 polysialic acid capsule (Stummeyer et al., 2006; Schwarzer et al., 2007; Buth et al., 2018). The T4-like phage had three different alleles of this protein, one found in HP3 and HP3.1, one found in ES12 and ES26, and one found in ES19 and ES21 (Supplementary Figure 2 and Supplementary Table 3). Interestingly, this gene is not found in phage T4. Analysis of the annotated domains also uncovered another protein not found in phage T4 and predicted to contain a lytic transglycosylase domain, which can have N-acetylmuramidase activity (Fukushima et al., 2008). ES17 was also found to contain a putative transglycosylase domain. Interestingly, the putative endolysin and the putative transglycosylase domain identified in phage ES17 were unlike anything found in either the T4-like phage or the literature.

### Anti-biofilm Phage Cocktail Synergizes With Antibiotics in Human Urine

**FIGURE 6:**
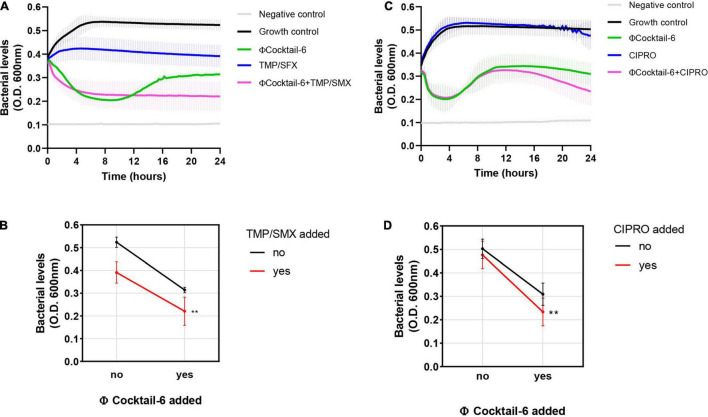
Phage cocktail-antibiotic interactions in human urine. Bacterial growth was monitored for 24 h by measuring OD_600_ in the presence of different antimicrobial combinations. Growth curves and interaction plots show additive effects of representative phage-antibiotic combinations: **(A,B)** trimethoprim/sulfamethoxazole (TMP/SMX), 8/152 μg/mL plus 10^8^ PFU/mL of ΦCocktail-6; **(C,D)** ciprofloxacin (CIPRO), 8 μg/mL plus 10^9^ PFU/mL of ΦCocktail-6. ΦCocktail-6: HP3, ES12, ES17, ES19, ES21, and ES26. **p < 0.01.

Although phage may eventually be a stand-alone treatment, previous studies suggest that using phage and antibiotics together may be advantageous (Khawaldeh et al., 2011; Aslam et al., 2020; Gu Liu et al., 2020). We determined the effect of multiple ΦCocktail-6 titers in combination with increasing concentrations of trimethoprim/sulfamethoxazole (sensitive) and ciprofloxacin (resistant) on the growth DS515 in urine (data not shown). DS515 treated with trimethoprim/sulfamethoxazole (8/152 μg/mL) resulted in ∼50% of the bacterial killing achieved by the ΦCocktail-6 alone (10^8^ PFU/mL) (Figure 6A). Resistance to the phage cocktail emerged at 12 h post-treatment in the phage alone treatment but was not observed in the presence of the antibiotic. Combined phage-antibiotic treatment resulted in higher bacterial killing compared to any of the antimicrobial treatments alone (Figure 6B). Finally, we assessed the effect of combining ΦCocktail-6 with ciprofloxacin to which DS515 is resistant. As expected, treatment with ciprofloxacin alone did not impair bacterial growth, whereas treatment with phage cocktail (10^9^ PFU/mL) resulted in ∼50% reduction in bacterial levels (Figure 6C). Resistance to phage emerged at around 6 h post-treatment with 10^9^ PFU/mL of ΦCocktail-6. Interestingly, combined phage-antibiotic treatment resulted in higher bacterial killing at 24 h suggesting that phage treatment re-sensitized the bacterial cells to a previously resistant antibiotic (Figures 6C,D).

### Anti-biofilm Phage Cocktails Reduce 7-Day-Old Biofilms and Biofilms on Catheter Material in Human Urine

**FIGURE 7:**
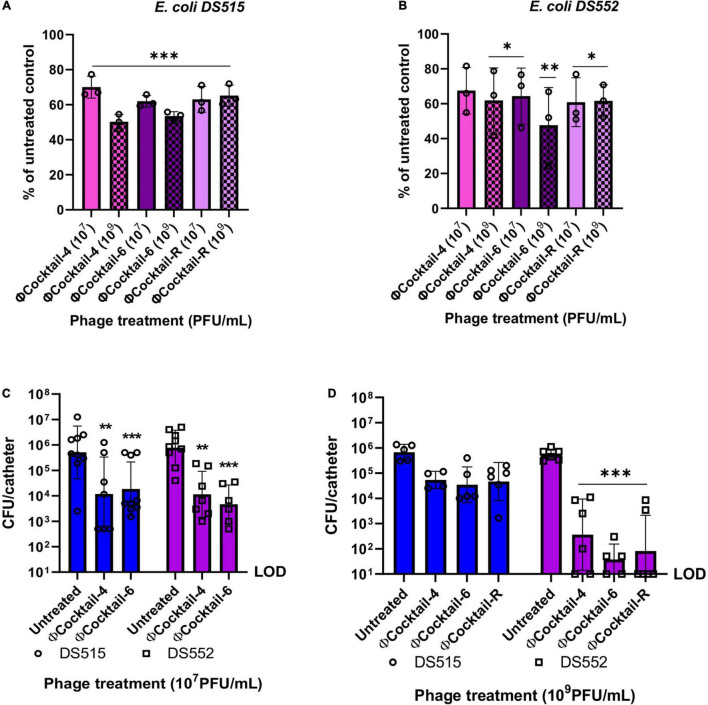
Activity of phage cocktails against 7-day biofilms and biofilms on catheter material in human urine. Biofilms of *E. coli* DS515 **(A)** and DS552 **(B)** were grown in human urine for seven consecutive days and then treated with two doses of phage cocktails for 24 h. Results depict the percentage of viable biofilms after treatment with phage, using the untreated control biofilm as the denominator **(A,B)**. Silicon RenaSil tubing pieces (∼6 mm) were incubated in bacterial suspensions in human urine for 48 h, washed and treated with 10^7^ PFU/mL of phage cocktails for 4 h **(C)** or 10^9^ PFU/mL of phage cocktails for 6 h **(D)**. Silicon tubing was washed, sonicated, and the resulting fluid was plated to quantify the bacterial burden. ΦCocktail-4: HP3, ES17, ES21, and ES26, ΦCocktail-6: HP3, ES12, ES17, ES19, ES21, and ES26, and ΦCocktail-R: HP3, ES17, ES19, and HP3.1. *p < 0.05, ^**^p < 0.01, ^***^p < 0.001. CFU, colony forming units; LOD, limit of detection.

Susceptibility of biofilms to phage activity may vary depending on the age of the biofilm (Verma et al., 2010; Vidakovic et al., 2018). To determine the efficacy of our phage cocktails against biofilms in an older developmental stage, *E. coli* DS515 and DS552 biofilms grown in human urine for seven consecutive days were treated with phage for 24 h. Biofilms on microplates were exposed to ΦCocktail-4, ΦCocktail-6 and ΦCocktail-R (10^7^ PFU/mL and 10^9^ PFU/mL) and viability of the remaining biofilm was determined using an MTT assay. One biological replicate per condition was plated on BD™ CHROMagar™ orientation medium to confirm that only *E. coli* was present in the biofilms at the end of the experiment. Treatment with phage cocktails resulted in reductions of 30–52.3% in biofilm viability for both bacterial strains (Figures 7A,B). Treatment with 10^9^ PFU/mL of cocktail ΦCocktail-4 resulted in the highest reduction in biofilm viability for *E. coli* DS515 (49.9%), whereas treatment with 10^9^ PFU/mL of ΦCocktail-6 resulted in the highest reduction in biofilm viability for *E. coli* DS552 (52.3%).

Finally, we explored the efficacy of our novel phage cocktails against *E. coli* biofilms on RenaSil silicon tubing (silicone urinary catheters predominate in the clinical setting) in urine. Pre-formed 48-h biofilms of *E. coli* DS515 and DS552 on silicon tubing were treated with phage cocktails for 4 h. The number of live cells within the biofilm after phage treatment (10^7^ PFU/mL) was quantified by determining colony forming units. Treatment with ΦCocktail-4 and ΦCocktail-6 resulted in a decrease in bacterial burden of ∼2 logs for both strains (Figure 7C). Treating biofilms of *E. coli* DS515 for 6 h with a higher dose of phage cocktails (10^9^ PFU/mL) resulted in no significant decrease in bacterial burdens (Figure 7D). This may be due to the ability of DS515 to recover after initial phage suppression as observed in Figure 3A. As previously observed (Figure 5A), the presence of phage HP3.1 in ΦCocktail-R did not help to overcome the tolerance of DS515 to the phage cocktails (Figure 7D), suggesting that different mechanisms of resistance to those targeted by phage HP3.1 are involved in the inability of phage to reduce viability of DS515 biofilms observed under these conditions. In contrast, increasing the dose of phage cocktails and incubation time resulted in a higher reduction (∼4-log) in bacterial burdens recovered from *E. coli* DS552 biofilms incubated with all cocktails tested (Figure 7D).

## Discussion

Biofilm formation is a key virulence determinant that facilitates *E. coli* pathogenesis in the urinary tract and is associated with increased fitness in the bladder, and higher rates of antimicrobial resistance and relapse after the original UTI (Soto et al., 2006; Sanchez et al., 2013; Karigoudar et al., 2019; Shah et al., 2019). In this study, we developed and characterized a novel strategy to combat *E. coli* biofilms. We found that: (i) biofilm formation by individual *E. coli* strains differs in complex medium and urine, (ii) some phage are lytic and maintain biofilm-killing capabilities in urine, (iii) cocktails of phage from our library target most *E. coli* isolated from our patient population, (iv) phage cocktails with broad host range are highly effective in two models of CAUTI, and (v) phage cocktails synergize with antibiotics.

Our results shed light on the biofilm production phenotypes of *E. coli* strains isolated from the urine of patients with SCI, who frequently require chronic bladder catheterization (Hines-Munson et al., 2021). Virtually all *E. coli* strains in our library formed biofilms. Interestingly, biofilm formation by individual strains varied depending on the growth medium, with some strains forming more biofilm in TSB compared with urine and vice versa. This is not surprising as the expression of adhesins and other structural components of biofilms is affected by environmental conditions, including the availability of nutrients and oxygen concentration (Hufnagel et al., 2016; Eberly et al., 2017). Additionally, differences in function of type 1 pili, which are critical for biofilm formation and colonization of the bladder in mouse models, have been found in *E. coli* isolated from urine despite the presence of intact *fim* operons (Schreiber et al., 2017).

Here, we identified a group of phage that reduce biofilms of two distinct *E. coli* isolates. Many phage with anti-biofilm abilities produce plaques with halos on bacterial lawns. These include phage ES17 which encodes a putative capsular depolymerase (pectinesterase) within its tail fibers (Green et al., 2021). The production of plaques with halos is associated with the degradation of the surrounding cells and/or their matrix material by phage-encoded depolymerase enzymes and increased performance against biofilms (Pires et al., 2016). Some phage with anti-biofilm activity did not produce plaques with halos (for example, HP3 and 6950). Physical properties including phage adsorption and amplification rates and phage diffusion within biofilms, are predicted to impact degradation of biofilms by phage (Gonzalez et al., 2018; Simmons et al., 2018; Hansen et al., 2019). These may play a role in the lytic activity against biofilms observed in phage that do not produce halos.

Traditionally, therapeutic phage are identified by determining phage lysis of planktonic cells or bacterial lawns (Hyman, 2019). Here we observed that efficient lysis of bacterial lawns or biofilms did not always translate into efficient lysis of planktonic cells in urine, which seemed in part to be dependent on the bacterial strain tested. Consistent with reports that phage are lytic in human environments such as blood (Ma et al., 2018), urine (Valerio et al., 2017) and mucosal surfaces (Green et al., 2021), we show that anti-biofilm phage are lytic in urine, but their activity may differ depending on the bacterial growth conditions. This highlights the importance of testing phage activity under host relevant conditions to unravel potential limitations that individual phage may have to kill the target strain. This type of sensitivity testing would increase the quality of therapeutic phage. Indeed, the inability of therapeutic phage to reduce biofilms has been hypothesized to have caused therapy failure in cases of chronic biofilm-associated infections (Aslam et al., 2020).

One of the limiting steps in developing therapeutic phage is identifying phage that are specific for the bacterial strain causing the infection. To overcome this, we created cocktails that target most strains in our *E. coli* library. Phage in our cocktails synergized to effectively reduce biofilms in two *in vitro* models of CAUTI. Of particular significance is the effectiveness of phage cocktails at low concentrations against biofilms formed on 96-well plates, the ∼2 log decrease in biofilms on silicon catheter material after only 4 h of phage treatment at a medium dose, and the ∼4 log decrease in DS552 biofilms on catheter material when the phage dose and incubation period is increased. The higher effectiveness of cocktails compared to individual phage may be due to the collective behaviors of the group, such as exploitation of the activity of depolymerase enzymes produced by some phage in the cocktail (for example ES17) by other phage that do not produce these types of enzymes (Cornelissen et al., 2011; Schmerer et al., 2014).

Our bioinformatic analysis showed that HP3/HP3.1, ES12, ES19, ES21, and ES26 encode a distal long-tail fiber protein with putative endosialidase activity that may target extracellular bacterial polysaccharides. Three distinct alleles of the putative long-tail fiber protein were identified, which may explain the differences in host-range and plaque morphologies among these phage as it has been reported for other coliphage (Stummeyer et al., 2006; Guo et al., 2017). For example, while phage ES19 and ES21 produced plaques with halos, phage HP3 and HP3.1 encoding a different allele of the same protein did not produce plaques with halos and showed lower activity against biofilms relative to the other T4-like phage.

We also demonstrated that our phage cocktails maintain anti-biofilm activity against older biofilms grown in human urine. The activity of individual phage has been shown to decrease in older biofilms (Vidakovic et al., 2018). Our findings reveal another advantage for the potential use of phage combinations to treat long-term biofilms associated with CAUTI. Previous studies have reported the effectiveness of phage to reduce biofilm formation on catheter material (Curtin and Donlan, 2006; Fu et al., 2010; Townsend et al., 2020; Rakov et al., 2021), however, the effect of urine on phage activity in these model systems had not been explored. Our results are very promising in that phage cocktails may reduce or eliminate biofilms on silicon medical devices *in vivo* and this is currently being evaluated by our group.

Phage may find a therapeutic niche as adjunct therapy to be given in addition to antibiotics. We observed additive effects between ΦCocktail-6 and two antibiotics commonly used to treat UTI (trimethoprim/sulfamethoxazole and ciprofloxacin) irrespective of the sensitivity status of *E. coli* DS515. As it has been shown with combination of phage HP3 and antibiotics (Gu Liu et al., 2020), the combination of ΦCocktail-6 and antibiotics decreased the bacterial revival observed at late time-points in phage-alone treatments. The addition of trimethoprim/sulfamethoxazole completely inhibited the emergence of phage-resistant cells, whereas increased sensitivity to ciprofloxacin was observed in cells that grew after initial suppression. It is hypothesized that the simultaneous or sequential exposure of bacterial cells to two selective pressures (phage and antibiotics) decreases the chances of emergence of resistance (Torres-Barcelo and Hochberg, 2016). Tradeoffs of developing resistance to one agent may increase sensitivity to the second agent (Verma et al., 2009; Burmeister et al., 2020; Burmeister and Turner, 2020), which may be the mechanism at play when DS515 is treated with ΦCocktail-6 and ciprofloxacin. The present study supports the advantages of using combinatorial treatments of phage and antibiotics that have been previously described *in vitro* and *in vivo* (Torres-Barcelo and Hochberg, 2016; Suh et al., 2022).

Although testing antimicrobials under clinically relevant conditions (for example human urine) may be advantageous, there are some limitations to this approach. Bacterial growth is limited under the nutrient restricted conditions in urine, and there are host components that are not present *in vitro*, which potentially aid bacterial growth during UTI, including proteins such as fibrinogen that are induced during inflammation (Flores-Mireles et al., 2019). Thus, evaluation of phage-antibiotic interactions in additional growth media may be warranted. A limitation of our study is that phage-antibiotic synergy against biofilms was not explored. The addition of phage decreased antibiotic concentrations required to eradicate *E. coli* biofilms in a study by Hyman (2019). As we observed, phage killing of biofilms and planktonic cells in urine can differ greatly, thus results of phage-antibiotic synergy in planktonic cells may not be completely translatable to biofilms. Our future studies will evaluate phage-antibiotic interactions during treatment of *E. coli* biofilms.

Creating a phage cocktail active against contemporary *E. coli* isolates from patients with SCI at our institution circumvents many of the barriers to initiating clinical evaluation of phage as a therapeutic option to recalcitrant CAUTI. Since the phage included in our phage mixtures have been extensively characterized, and some have also been successfully used for phage therapy in animal models of infection and in a human case of compassionate use (Green et al., 2017; Aslam et al., 2020; Terwilliger et al., 2021), we believe that these anti-biofilm phage cocktails have high therapeutic potential to treat CAUTI. Future studies will evaluate the efficacy, pharmacokinetics and pharmacodynamics of the cocktails described here in animal models of CAUTI and will allow further refinement of our phage cocktails before clinical evaluation.

## Data Availability Statement

The raw data supporting the conclusions of this article will be made available by the authors, without undue reservation.

## Author Contributions

BS, EH, and SG performed the experiments and analyzed the data. JC performed the bioinformatic analysis of depolymerase enzymes. BT, FS, KM, and CH-M arranged for the collection of clinical isolates and corresponding antibiotic sensitivity data. RR and HK contributed to the design of the study and edited the manuscript. BS designed the study and wrote the manuscript. BT and AM contributed to the design and overall major goals of the study and edited the manuscript. All authors contributed to the article and approved the submitted version.

## Conflict of Interest

BT has received research support from Genentech for COVID trials. The remaining authors declare that the research was conducted in the absence of any commercial or financial relationships that could be construed as a potential conflict of interest.

## Publisher’s Note

All claims expressed in this article are solely those of the authors and do not necessarily represent those of their affiliated organizations, or those of the publisher, the editors and the reviewers. Any product that may be evaluated in this article, or claim that may be made by its manufacturer, is not guaranteed or endorsed by the publisher.

## Footnotes

1
https://www.geneious.com

## Supplementary Material

Supplementary Figure 1 Activity of phage cocktails against *E. coli* isolated from the urine of patients with SCI. Viability of cells in the biofilm under each condition after 24-h treatment with 10^7^ PFU/mL of phage cocktails is represented as a percentage of the untreated control. Biofilms of *E. coli* DS457, DS517 and DS549 in TSB **(A)** and human urine **(B)** are shown. ^***^*p* < 0.001, ^**^*p* < 0.01, **p* < 0.05.

Supplementary Figure 2 MAFFT alignment of the amino acid sequence of the distal tail-fiber protein with putative endosialidase activity identified in phage included in the anti-biofilm phage cocktails. An identity histogram is shown at the top: green represents 100% identity across samples, greeny-brown represents between 30 and 100% identity, and red represents <30% identity. Black lines represent amino acid differences from the majority consensus.
