# Corrigendum: Development of phage cocktails to treat *E. coli* catheter-associated urinary tract infection and associated biofilms

**DOI:** 10.3389/fmicb.2022.953136

**Published:** 2022-08-09

**Authors:** Belkys C. Sanchez, Emmaline R. Heckmann, Sabrina I. Green, Justin R. Clark, Heidi B. Kaplan, Robert F. Ramig, Kenneth L. Muldrew, Casey Hines-Munson, Felicia Skelton, Barbara W. Trautner, Anthony W. Maresso

**Affiliations:** ^1^Tailored Antibacterials and Innovative Laboratories for Phage (Φ) Research, Department of Molecular Virology and Microbiology, Baylor College of Medicine, Houston, TX, United States; ^2^Department of Microbiology and Molecular Genetics, McGovern Medical School, UTHealth Houston, Houston, TX, United States; ^3^Department of Pathology and Immunology, Baylor College of Medicine, Houston, TX, United States; ^4^Pathology and Laboratory Medicine, Michael E. DeBakey VA Medical Center, Houston, TX, United States; ^5^Section of Infectious Diseases, Department of Medicine, Baylor College of Medicine, Houston, TX, United States; ^6^Center for Innovations in Quality, Effectiveness and Safety, Michael E. DeBakey VA Medical Center, Houston, TX, United States; ^7^H. Ben Taub Department of Physical Medicine and Rehabilitation, Baylor College of Medicine, Houston, TX, United States; ^8^Department of Medicine and Surgery, Baylor College of Medicine, Houston, TX, United States; ^9^Center for Translational Research on Inflammatory Diseases, Michael E. DeBakey VA Medical Center, Houston, TX, United States

**Keywords:** phage therapy, uropathogenic *E. coli*, multidrug-resistance, CAUTI, biofilms

In the published article **“Kenneth L. Muldrew”** was not included as an author. The corrected Author Contributions Statement appears below.

BS, EH, and SG performed the experiments and analyzed the data. JC performed the bioinformatic analysis of depolymerase enzymes. BT, FS, KM, and CH-M arranged for the collection of clinical isolates and corresponding antibiotic sensitivity data. RR and HK contributed to the design of the study and edited the manuscript. BS designed the study and wrote the manuscript. BT and AM contributed to the design and overall major goals of the study and edited the manuscript. All authors contributed to the article and approved the submitted version.

The authors apologize for this error and state that this does not change the scientific conclusions of the article in any way. The original article has been updated.

## Publisher's note

All claims expressed in this article are solely those of the authors and do not necessarily represent those of their affiliated organizations, or those of the publisher, the editors and the reviewers. Any product that may be evaluated in this article, or claim that may be made by its manufacturer, is not guaranteed or endorsed by the publisher.

