# Supplementary material for: Development of Phage Cocktails to Treat E. coli Catheter-Associated Urinary Tract Infection and Associated Biofilms
Source: Front Microbiol. 2022 May 10;13:796132. doi: 10.3389/fmicb.2022.796132 (PMC9127763; doi:10.3389/fmicb.2022.796132)
Supplement: Supplementary file 1 [file Data_Sheet_1.zip › Table S1.docx]

| **Table S1: Properties of phages used in biofilm screen** | | | | | | |  |  |
| --- | --- | --- | --- | --- | --- | --- | --- | --- |
| **Phage name** | **Source** | **Level of DS515 biofilm killing (10^7^PFU/mL)** | **Anti-biofilm phage group** | **Halo** | **Clears DS515 lawns** | **Reference** |  |  |
| ES26 | Human sewage | >50% | 1 | Yes | Yes | Gibson, 2019 |  |  |
| HP3 | Goose & Duck feces | >50% | 1 | No | Yes | Green, 2017 |  |  |
| 6914 | Human sewage | >50% | 1 | No | Yes | TAILΦR^a^ |  |  |
| 6936 | Human sewage | >50% | 1 | No | Yes | TAILΦR^a^ |  |  |
| MX4 | Human sewage | >50% | 1 | Yes | Yes | This study |  |  |
| ES17 | Human sewage | >50% | 1 | Yes | Yes | Gibson, 2019 |  |  |
| MX1 | Human sewage | >50% | 1 | Yes | Yes | This study |  |  |
| MX3 | Human sewage | >50% | 1 | Yes | Yes | This study |  |  |
| MX2 | Human sewage | 25-50% | 2 | Yes | Yes | This study |  |  |
| CI4 | - | 25-50% | 2 | No | Yes | ^b^ |  |  |
| 6950 | Human sewage | 25-50% | 2 | No | Yes | TAILΦR^a^ |  |  |
| 6954 | Human sewage | 25-50% | 2 | No | Yes | TAILΦR^a^ |  |  |
| 6915 | Human sewage | 25-50% | 2 | Yes | Yes | TAILΦR^a^ |  |  |
| 6948 | Human sewage | 25-50% | 2 | Yes | Yes | TAILΦR^a^ |  |  |
| 6955 | Human sewage | 25-50% | 2 | No | Yes | TAILΦR^a^ |  |  |
| HP3.1 | Evolved from HP3 | 20% | 3 | No | Yes | Gibson, 2019 |  |  |
| 6949 | Human sewage | 19% | 3 | No | Yes | TAILΦR^a^ |  |  |
| CF2 | Chicken feces | ns | 3 | No | No | Green, 2017 |  |  |
| CI5 | - | ns | 3 | No | Yes | ^b^ |  |  |
| CG1 | Human sewage | ns | 3 | Yes | Yes | This study |  |  |
| CG3 | Human sewage | ns | 3 | Yes | Yes | This study |  |  |
| ES12 | Human sewage | ns | 3 | No | Yes | Gibson, 2019 |  |  |
| EC1 | Dog feces | ns | 3 | No | No | Green, 2017 |  |  |
| 6935 | Human sewage | ns | 3 | - | No | TAILΦR^a^ |  |  |
| CG2 | Human sewage | ns | 3 | Yes | Yes | This study |  |  |
| ES21 | Human sewage | ns | 3 | Yes | Yes | Gibson, 2019 |  |  |
| ES19 | Human sewage | ns | 3 | Yes | Yes | Gibson, 2019 |  |  |
| 6925 | Human sewage | ns | 3 | No | Yes | TAILΦR^a^ |  |  |
| ^a^ Phages obtained from Tailored Antibacterials and Innovative Laboratories for Phage (Φ) Research Laboratories (TAILΦR) | | | | | | |  |  |
| ^b^ Phages obtained from the TAILΦR library and originally isolated by Andrey Filippov Lab at Walter Reed Army Institute of Research  ns: not statistically significant | | | | | | |  |  |
