# Supplementary material for: Development of Phage Cocktails to Treat E. coli Catheter-Associated Urinary Tract Infection and Associated Biofilms
Source: Front Microbiol. 2022 May 10;13:796132. doi: 10.3389/fmicb.2022.796132 (PMC9127763; doi:10.3389/fmicb.2022.796132)
Supplement: Supplementary file 1 [file Data_Sheet_1.zip › Table S2.docx]

| **Table S2:** Characteristics of *E. coli* clinical isolates from patients with spinal cord injury at the Houston VA Hospital | | | | | | | | | | | | | | | | | | | | |
| --- | --- | --- | --- | --- | --- | --- | --- | --- | --- | --- | --- | --- | --- | --- | --- | --- | --- | --- | --- | --- |
| **Strain ID** | **Date collected** | **Antibiotic sensitivity** | | | | | | | | | | | | | **Phage sensitivity (EOP)** | | | | | |
|  |  | **Amikacin** | **Ampicillin** | **Cefepime** | **Gentamicin** | **Imipenem** | **Levofloxacin** | **Pip/Tazobactam** | **Amp/Sulbactam** | **Ceftriaxone** | **Cefazolin** | **Nitrofurantoin** | **Ertapenem** | **TMP/SMZ** | **φHP3** | **φES12** | **φES17** | **φES19** | **φES21** | **φES26** |
| **DS452^##^** | 10/23/18 |  |  |  |  |  |  | **NT** |  |  |  |  |  |  | **-** |  |  | **-** | **-** |  |
| **DS453^##^** | 10/24/18 |  |  |  |  |  |  |  |  |  |  |  |  |  |  | **-** |  |  |  |  |
| **DS454^##^** | 11/01/18 |  |  |  |  |  |  |  |  |  |  |  |  |  | **-** | **-** | **-** | **-** | **-** | **-** |
| **DS455^##^** | 10/31/18 |  |  |  |  |  |  |  |  |  |  |  |  |  | **-** | **-** | **-** | **-** | **-** | **-** |
| **DS456^##^** | 11/05/18 |  |  |  |  |  |  |  |  |  |  |  |  |  |  | **-** | **-** | **+** | **+** | **-** |
| **DS457^##^** | 11/05/18 |  |  |  |  |  |  |  |  |  |  |  |  |  |  | **+** |  | **+** |  | **+** |
| **DS458^##^** | 11/13/18 |  |  |  |  |  |  |  |  |  |  |  |  |  | **-** | **-** | **-** | **-** | **-** | **-** |
| **DS459^##^** | 11/19/18 |  |  |  |  |  |  |  |  |  |  |  |  |  |  |  | **-** |  |  |  |
| **DS460^##^** | 12/05/18 |  |  |  |  |  |  |  |  |  |  |  |  |  |  | **-** | **-** | **+** | **+** |  |
| **DS461^##^** | 12/11/18 |  |  |  |  |  |  |  |  |  |  |  |  |  |  |  | **-** | **+** | **+** |  |
| **DS462^##^** | 12/17/18 |  |  |  |  |  |  |  |  |  |  |  |  |  |  | - | **+** | - |  |  |
| **DS463^##^** | 12/18/18 |  |  |  |  |  |  |  |  |  |  |  |  |  |  | - | - | **+** | **+** | - |
| **DS473** | 02/27/19 |  |  |  |  |  |  | **NT** |  |  |  |  |  |  | **-** | **-** |  | **-** |  | **-** |
| **DS481** | 03/27/19 |  |  |  |  |  |  |  |  |  |  |  |  |  |  | **-** | **-** |  | **+** | **-** |
| **DS483** | 04/03/19 |  |  |  |  |  |  |  |  |  |  |  |  |  |  |  | **-** |  |  |  |
| **DS493** | 04/16/19 |  |  |  |  |  |  |  |  |  |  |  |  |  |  | **-** | **-** |  | **+** | **-** |
| **DS495** | 04/17/19 |  |  |  |  |  |  |  |  |  |  |  |  |  |  | **+** | **-** |  |  |  |
| **DS499** | 04/23/19 |  |  |  |  |  |  |  |  |  |  |  |  |  |  | **+** | **-** | **+** | **+** |  |
| **DS503** | 05/01/19 |  |  |  |  |  |  |  |  |  |  |  |  |  |  | **+** | **-** |  |  |  |
| **DS508** | 04/29/19 |  |  |  |  |  |  |  |  |  |  |  |  |  |  |  |  |  | **+** |  |
| **DS510** | 05/15/19 |  |  |  |  |  |  |  |  |  |  |  |  |  |  | **-** |  | **-** | **-** | **-** |
| **DS513** | 05/10/19 |  |  |  |  |  |  |  |  |  |  |  |  |  |  |  |  |  |  |  |
| **DS515** | 05/10/19 |  |  |  |  |  |  |  |  |  |  |  |  |  |  | **+** |  |  | **+** |  |
| **DS516** | 05/15/19 |  |  |  |  |  |  |  |  |  |  |  |  |  |  |  | **-** | **+** | **+** |  |
| **DS517** | 05/16/19 |  |  |  |  |  |  |  |  |  |  |  |  |  |  | **+** | **-** | **+** | **+** |  |
| **DS518** | 05/22/19 |  |  |  |  |  |  |  |  |  |  |  |  |  |  | **-** |  | **-** | **-** | **-** |
| **DS528** | 05/24/19 |  |  |  |  |  |  |  |  |  |  |  |  |  |  | **-** | **-** |  | **+** | **-** |
| **DS531** | 06/03/19 |  |  |  |  |  |  |  |  |  |  |  |  |  |  | **-** |  | **-** |  |  |
| **DS535** | 06/18/19 |  |  |  |  |  |  |  |  |  |  |  |  |  |  |  | **-** | **+** | **+** |  |
| **DS540** | 07/01/19 |  |  |  |  |  |  | **NT** |  |  |  |  |  |  | **-** |  | **-** |  |  |  |
| **DS544** | 07/01/19 |  |  |  |  |  |  |  |  |  |  |  |  |  |  |  | **-** |  |  |  |
| **DS549** | 07/09/19 |  |  |  |  |  |  |  |  |  |  |  |  |  |  | **+** | **+** | **+** |  | **+** |
| **DS552** | 07/17/19 |  |  |  |  |  |  |  |  |  |  |  |  |  |  |  | **-** | **+** | **+** |  |
| **DS559** | 07/24/19 |  |  |  |  |  |  |  |  |  |  |  |  |  |  |  | **+** |  |  |  |
| **DS564** | 07/30/19 |  |  |  |  |  |  |  |  |  |  |  |  |  | **-** | **-** | **-** | **-** | **-** | **-** |
| **DS566** | 08/06/19 |  |  |  |  |  |  | **NT** |  |  |  |  |  |  |  |  | **-** | **+** |  |  |
| **DS570** | 08/20/19 |  |  |  |  |  |  |  |  |  |  |  |  |  | **-** | **-** | **-** | **-** | **-** | **-** |
| **DS572** | 08/19/19 | **NT** |  |  |  |  |  |  |  |  |  |  |  |  |  | **-** | **-** | **-** | **-** | **-** |
| **DS573** | 08/15/19 | **NT** |  |  |  |  |  |  |  |  |  |  |  |  | **-** | **-** | **-** | **-** | **-** | **-** |
| **DS574** | 08/16/19 | **NT** |  |  |  |  |  |  |  |  |  |  |  |  |  | **-** | **+** |  |  | **+** |
| **DS575** | 08/22/19 |  |  |  |  |  |  |  |  |  |  |  |  |  |  | **+** |  |  | **-** | **+** |
| **DS582** | 09/06/19 |  |  |  |  |  |  |  |  |  |  |  |  |  |  | **+** |  | **-** |  | **+** |
| **DS592** | 09/20/19 |  |  |  |  |  |  |  |  |  |  |  |  |  |  |  |  |  |  | **+** |
| **DS598** | 10/07/19 |  |  |  |  |  |  |  |  |  |  |  |  |  | **-** | **-** | **-** | **-** | **-** |  |
| **DS607** | 10/22/19 |  |  |  |  |  |  |  |  |  |  |  |  |  |  | **-** | **-** | **-** | **-** |  |
| **DS609** | 10/22/19 |  |  |  |  |  |  |  |  |  |  |  |  |  |  |  |  |  |  |  |
| **DS612** | 10/28/19 |  |  |  |  |  |  |  |  |  |  |  |  |  |  |  |  |  |  |  |
| **DS619** | 11/06/19 |  |  |  |  |  |  |  |  |  |  |  |  |  |  |  |  |  |  | **+** |
| **DS622** | 11/06/19 |  |  |  |  |  |  | **NT** |  |  |  |  |  |  |  | **-** | **-** | **-** | **-** | **-** |
| **DS625** | 11/04/19 |  |  |  |  |  |  |  |  |  |  |  |  |  |  | **-** |  | **-** |  |  |
| **DS627** | 11/12/19 |  |  |  |  |  |  |  |  |  |  |  |  |  |  |  |  |  |  | **+** |
| **DS628** | 11/13/19 |  |  |  |  |  |  |  |  |  |  |  |  |  | **-** | **-** | **-** | **-** | **-** | **-** |
| **DS629** | 11/13/19 |  |  |  |  |  |  |  |  |  |  |  |  |  |  | **-** |  |  | **-** |  |
| **Footnotes** | | **Key to Abx sensitivity** | | | | | | | | | **Key to Phage sensitivity** | | | | | | | | | |
| ^#^**^##^** Strains previously characterized by Gibson et al. (2019)  **EOP:** Efficiency of plating | |  | Sensitive | | | | | | | | **+** | EOP>1.000 | | | | | | | | |
|  |  |  | Intermediate | | | | | | | |  | EOP 0.100-1.000 | | | | | | | | |
|  |  |  | Resistant | | | | | | | |  | EOP 0.001-0.099 | | | | | | | | |
|  |  |  | | | | | | | | |  | EOP < 0.001: not useful | | | | | | | | |
|  |  |  |  |  |  |  |  |  |  |  | **-** | No Growth | | | | | | | | |
|  |  |  |  |  |  |  |  |  |  |  | EOP=Titer X / Titer Ref | | | | | | | | | |
