# Supplementary material for: Development of Phage Cocktails to Treat E. coli Catheter-Associated Urinary Tract Infection and Associated Biofilms
Source: Front Microbiol. 2022 May 10;13:796132. doi: 10.3389/fmicb.2022.796132 (PMC9127763; doi:10.3389/fmicb.2022.796132)
Supplement: Supplementary file 1 [file Data_Sheet_1.zip › Table S3.docx]

| **Table S3:** Results of bioinformatic analysis to identify putative depolymerase enzymes in anti-biofilm phage. | | | | |
| --- | --- | --- | --- | --- |
| **Phage** | **Putative protein** | **Accession number** | **Domain found** | **Amino acid sequence similarity** |
| HP3  HP3.1  ES12  ES17  ES19  ES21  ES26 | Baseplate hub central spike | cd00735 | Phage lysozyme E | 100% |
| HP3  HP3.1  ES19  ES21 | Endolysin | cd00735 | Phage lysozyme E | 100% |
| ES12  ES26 |  |  |  | S40N mutation |
| HP3  HP3.1 | Distal long-tail fiber protein | cd19958  PFAM13884 | Pyocin knob  Chaperone of endosialidase | 100% |
| ES12  ES26 |  |  |  | 100% |
| ES19  ES21 |  |  |  | 100% |
| HP3  HP3.1  ES12  ES19  ES21  ES26 | Lytic transglycosidase | PF01464  cd00254 | Transglycosylase  SLT domain | 100% |
| ES17 | Endolysin | NP_049736.1 | Phage lysin  Phage lysozyme | --- |
| ES17 | Lytic transglycosidase | cd00254 | Lytic transglycosidase | --- |
| ES17^a^ | Tail Fiber Protein | --- | Pectinesterase | --- |
| ^a^Described by Green et al. 2021 | | | | |
