# Supplementary figures and images for: Development of Phage Cocktails to Treat E. coli Catheter-Associated Urinary Tract Infection and Associated Biofilms
Source: Front Microbiol. 2022 May 10;13:796132. doi: 10.3389/fmicb.2022.796132 (PMC9127763; doi:10.3389/fmicb.2022.796132)

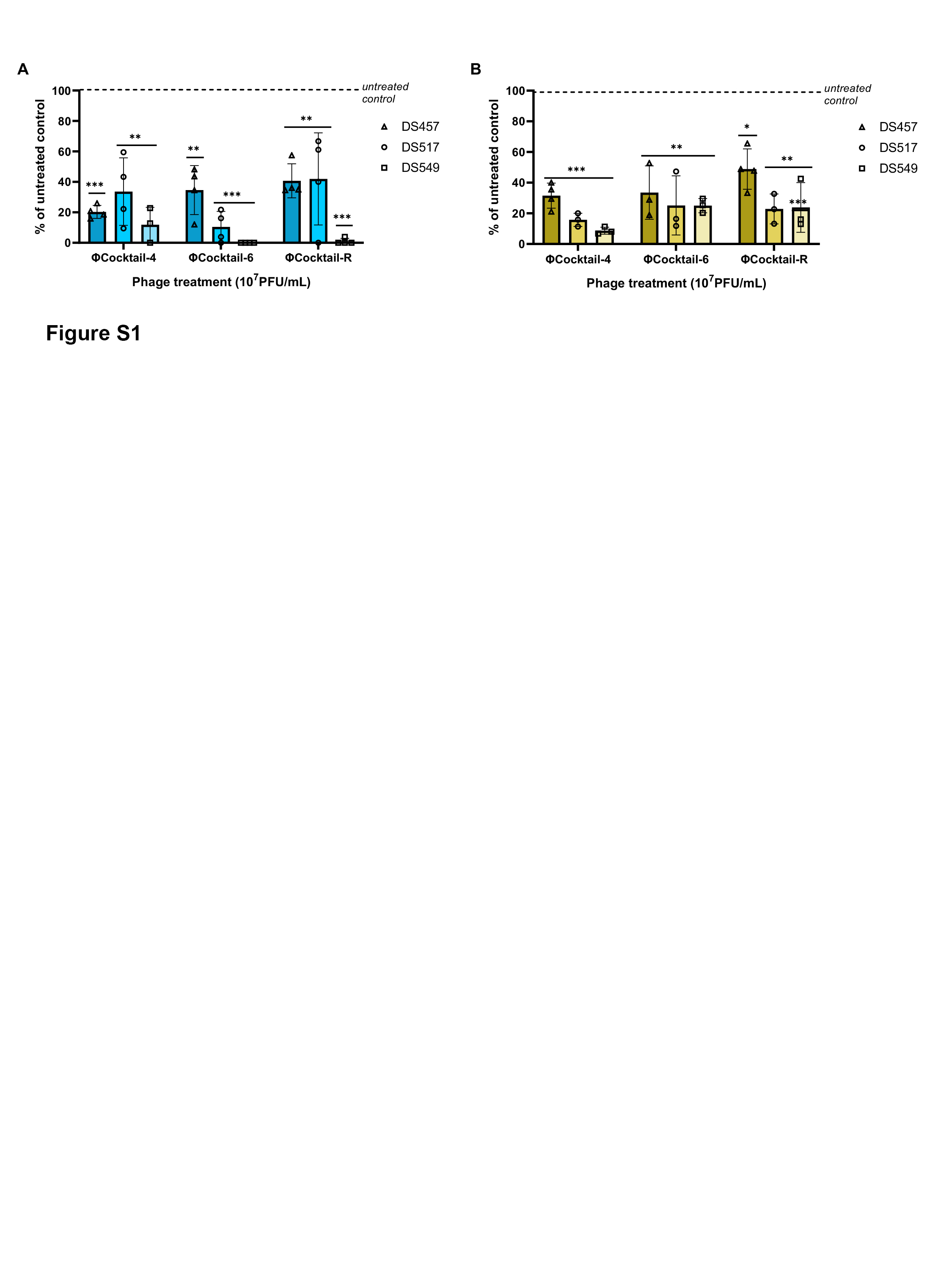

Supplement: Supplementary Figure 1 — Activity of phage cocktails against E. coli isolated from the urine of patients with SCI. Viability of cells in the biofilm under each condition after 24-h treatment with 107 PFU/mL of phage cocktails is represented as a percentage of the untreated control. Biofilms of E. coli DS457, DS517 and DS549 in TSB (A) and human urine (B) are shown. ***p < 0.001, **p < 0.01, *p < 0.05. [file Image_1.JPEG]

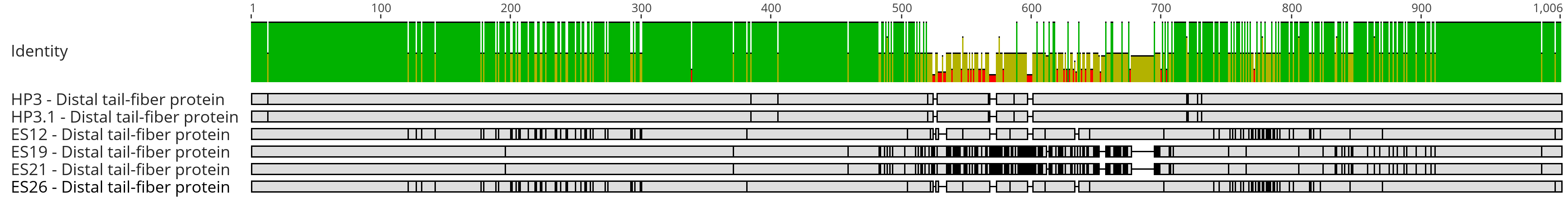

Supplement: Supplementary Figure 2 — MAFFT alignment of the amino acid sequence of the distal tail-fiber protein with putative endosialidase activity identified in phage included in the anti-biofilm phage cocktails. An identity histogram is shown at the top: green represents 100% identity across samples, greeny-brown represents between 30 and 100% identity, and red represents <30% identity. Black lines represent amino acid differences from the majority consensus. [file Image_2.JPEG]
